# 3D Super-Resolution Imaging of PSD95 Reveals an Abundance of Diffuse Protein Supercomplexes in the Mouse Brain

**DOI:** 10.1021/acschemneuro.4c00684

**Published:** 2024-12-20

**Authors:** Sam Daly, Edita Bulovaite, Anoushka Handa, Katie Morris, Leila Muresan, Candace Adams, Takeshi Kaizuka, Alexandre Kitching, Alexander Spark, Gregory Chant, Kevin O′Holleran, Seth G. N. Grant, Mathew H. Horrocks, Steven F. Lee

**Affiliations:** † Yusuf Hamied Department of Chemistry, University of Cambridge, Cambridge CB2 1EW, U.K.; ‡ Genes to Cognition Program, Centre for Clinical Brain Sciences, University of Edinburgh, Edinburgh EH16 4SB, U.K.; § RR Chemistry Hub, Institute for Regeneration and Repair, University of Edinburgh, Edinburgh EH16 4UU, U.K.; ∥ EaStCHEM School of Chemistry, University of Edinburgh, Edinburgh EH9 3FJ, U.K.; ⊥ Cambridge Advanced Imaging Centre, University of Cambridge, Cambridge CB2 3DY, U.K.; # ZOMP, Maxwell Centre, JJ Thomson Avenue, Cambridge CB3 0HE, U.K.; ∇ Lume VR Ltd., 26 Stanley Road, Oxford OX4 1QZ, U.K.

**Keywords:** super-resolution microscopy, PSD95, mouse brain, double helix PSF, 3D SMLM, clustering

## Abstract

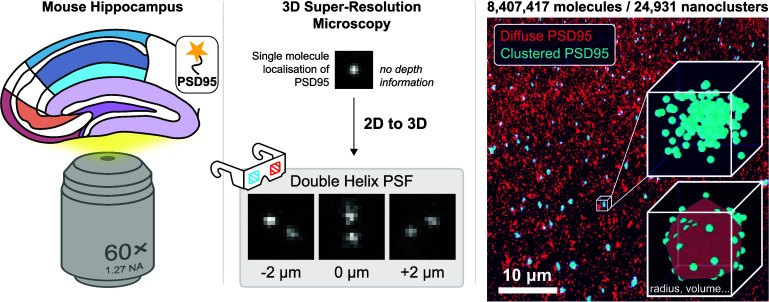

PSD95 is an abundant scaffolding protein that assembles multiprotein complexes controlling synaptic physiology and behavior. Confocal microscopy has previously shown that PSD95 is enriched in the postsynaptic terminals of excitatory synapses and two-dimensional (2D) super-resolution microscopy further revealed that it forms nanoclusters. In this study, we utilized three-dimensional (3D) super-resolution microscopy to examine the nanoarchitecture of PSD95 in the mouse brain, characterizing the spatial arrangement of over 8 million molecules. While we were able to identify molecular arrangements that have been previously reported, imaging in 3D allowed us to classify these with higher accuracy. Furthermore, 3D super-resolution microscopy enabled the quantification of protein levels, revealing that an abundance of PSD95 molecules existed outside of synapses as a diffuse population of supercomplexes, containing multiple copies of PSD95. Further analysis of the supercomplexes containing two units identified two populations: one that had PSD95 molecules separated by 39 ± 2 nm, and a second with a separation of 94 ± 27 nm. The finding that there exists supercomplexes containing two PSD95 units outside of the synapse suggests that supercomplexes containing multiple protein copies assemble outside the synapse and then integrate into the synapse to form a supramolecular nanocluster architecture.

## Introduction

Synapses are specialized junctions between nerve cells that contain thousands of proteins and underpin our understanding of cognition and learning.^1−5^ Most synaptic proteins are organized into multiprotein complexes that are essential for key functions such as synaptic transmission and plasticity.^6^ Biochemical studies confirm that scaffolding proteins assemble a variety of neurotransmitter receptors, ion channels, signaling proteins and transmembrane proteins into families of complexes in the postsynaptic terminal of excitatory synapses.^6,7^ Classical electron microscopy studies have shown an electron-dense structure beneath the postsynaptic membrane of excitatory synapses, known as the postsynaptic density (PSD).^8−10^ Recent studies employing cryogenic correlated light-electron microscopy (cryoCLEM) demonstrate that, rather than an increase in postsynaptic protein density, these molecules exhibit a specialized organizational structure in the postsynaptic terminal.^11^

The prototypical scaffolding protein of excitatory synapses is postsynaptic density protein 95 (PSD95), which plays a crucial role in synaptic transmission, synaptic plasticity and learning.^12^ PSD95 is highly abundant within synaptic protein extracts and found within 1–3 MDa complexes.^7,13,14^ Confocal microscopy of labeled endogenous PSD95 has confirmed its presence in the postsynaptic dendritic spine^15−17^ and super-resolution (SR) microscopy has explored the nanoscale architecture of PSD95, and other proteins in synaptic clusters, in cultured cells^18,19^ and brain tissue^20−22^. These studies revealed that approximately 100 PSD95 molecules were organized into nanoclusters (NCs)^14,20^ around 130 nm in size^20^ and located in the PSD, which varies in size from ∼100–500 nm in diameter.^20,23^ Receptors, including AMPARs have also been shown to form similar nanoclusters^19^ and their functional relevance has been demonstrated via their alignment to clusters at the presynapse, by so-called “nanocolumns”.^24^ The prevailing model suggests that PSD95 molecules participate in an assembly process where supercomplexes containing two copies of PSD95 are formed, which we have previously characterized.^25^ These supercomplexes then assemble into larger multiprotein complexes, which ultimately combine into NCs to form the PSD.^26^ However, it is still unclear whether all these assembly stages occur within the synapse itself, which limits our understanding of how synaptic structure and function are regulated both spatially and temporally.

To understand the organization and assembly of PSD95 into supramolecular structures, we have developed various genetic labeling strategies for SR microscopy.^20,21,27^ In particular, single-molecule localization microscopy (SMLM) has shown that synapses contain multiples of NCs (1 NC/PSD, 2 NC/PSD and 3+ NC/PSD) that are differentially distributed into synapses in regions of the hippocampal formation—a brain region important for learning and memory.^20^ However, this SMLM approach was limited to visualizing PSD95 in two dimensions and in regions close to the cutting plane of the fixed brain slices, which may have been subject to dissection artifacts.^28^ Furthermore, imaging biological samples in three-dimensional (3D) is crucial to avoid clustering artifacts that can distort quantitative imaging results and lead to inaccurate interpretations of spatial organization.

Various methodologies are available for studying protein distribution at the nanoscale, each offering different depth-of-field (DoF) and resolution specifications that depend on the sample being studied. These include multifocal plane approaches, such as biplane microscopy (<1 μm DoF),^29^ and point spread function (PSF) engineering techniques. PSF engineering methods include astigmatism (<1 μm DoF),^30^ the double-helix PSF (DHPSF, 2–4 μm DoF),^31^ the tetrapod PSF (4–8 μm DoF),^32,33^ and single-molecule light field microscopy (∼8 μm DoF).^34,35^ The DHPSF is well-suited to the investigation of sectioned tissue by affording greater resolution isotropy (*xy*: ∼ 10 nm and *z*: ∼ 20 nm)^36^ compared to astigmatism^37^ and a large ∼4 μm DoF that eliminates the need for axial scanning.^38^

We present the first application of DHPSF SR microscopy in tissue, enabling the most comprehensive study of the 3D organization of individual PSD95 molecules. We evaluated 8,407,417 single PSD95 proteins, and 24,931 NCs in 7 brain regions from 3 mouse brains. While the lower sensitivity of the DHPSF methodology led to lower apparent NC densities compared to previous two-dimensional (2D) SR microscopy studies, we observed comparable NC and PSD radii giving confidence in our cluster assignments in 3D. Furthermore, our results highlighted regional heterogeneity in PSD distribution that contain multiple NCs as observed previously with 2D SR imaging of PSD95 within synapses. Now with access to 3D spatial distributions of PSD95 in bulk tissue sections we reveal a previously undescribed subpopulation. The majority of PSD95 exists as a “diffuse” cloud of complexes containing multiple copies of the protein throughout the tissue volume, rather than being associated with the synapse.

## Results

### Volumetric SR Imaging of PSD95 NCs Using DHPSF Microscopy

**Figure 1:**
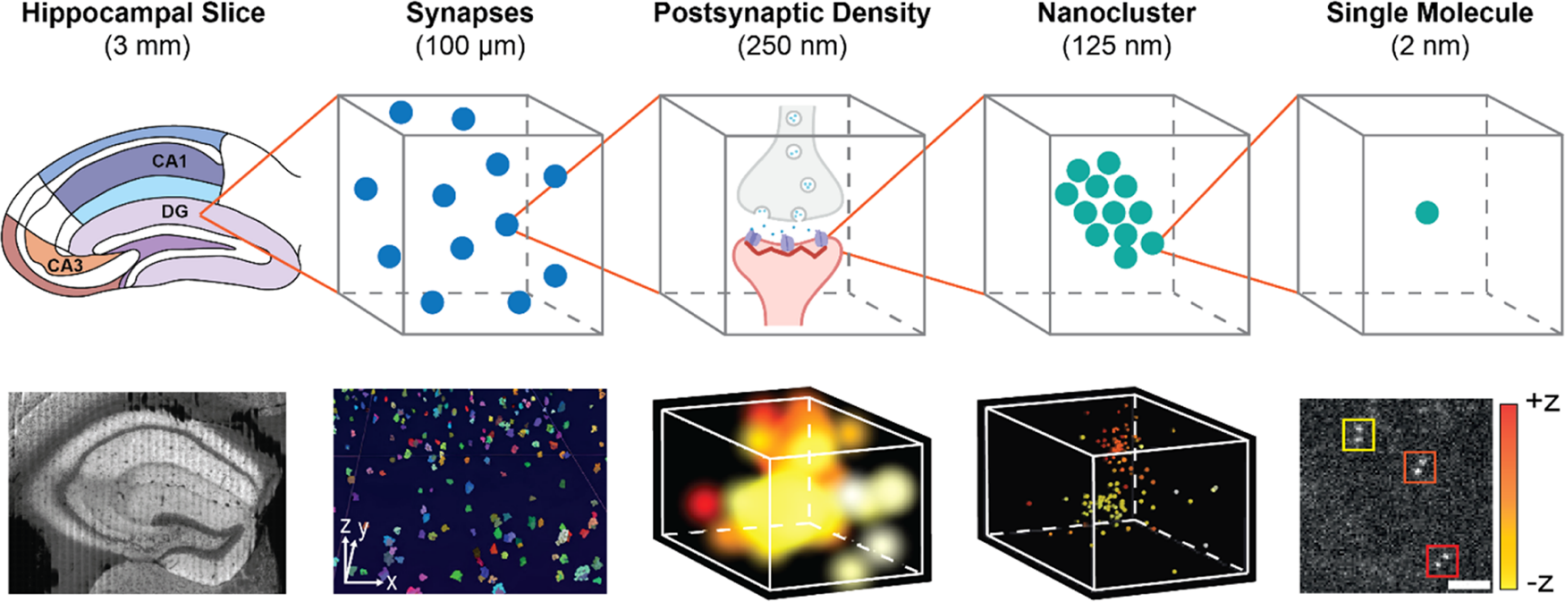
PSD95 Assembly at different spatial scales. (Top) schematic representations and relative sizes of the coronal hippocampus, a synapse, postsynaptic density (PSD), a nanocluster (NC) of PSD95, and a single mEos2-labeled PSD95 molecule. (Bottom) Raw data corresponding to the schematic shown above. Hippocampal slice: confocal image of a coronal hippocampus mouse brain slice. Synapses: CA1SR hippocampal region (30 μm × 30 μm × 4 μm) with ∼200 NCs; PSD: diffraction-limited image of PSD95 NCs; Nanocluster: super-resolved PSD95 NCs; single molecule: DHPSF SR microscopy data of a single PSD95 molecule fused to the fluorescent protein mEos2. Color indicates axial position and scale bar represents 5 μm.

**Figure 2:**
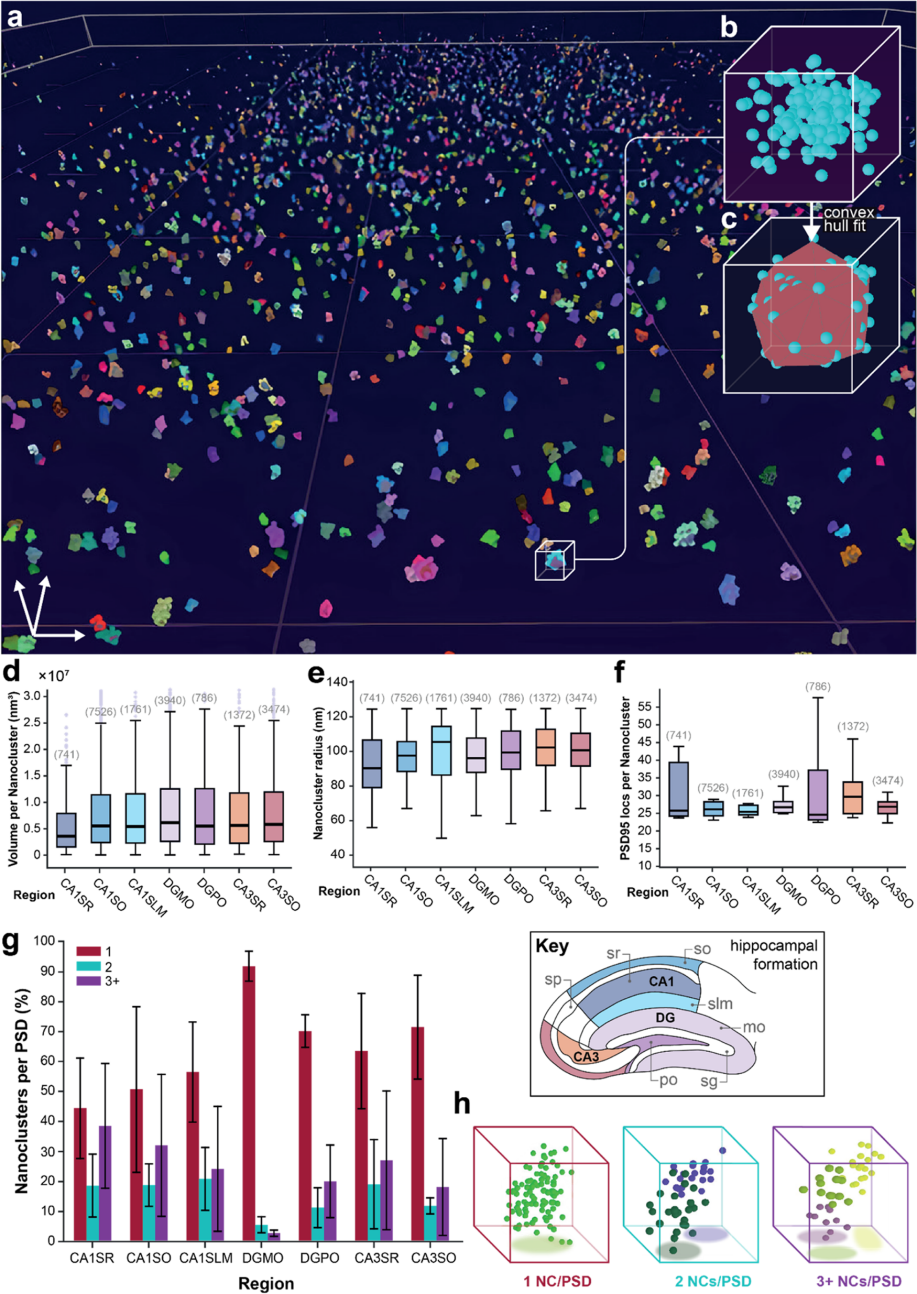
3D DHPSF imaging of PSD95 NCs. (a) Representative reconstruction of the CA1SR region (70 μm × 70 μm × 4 μm) with NCs represented as demarcated colors. (b) Expanded view of a NC with ∼30 localizations of PSD95. (c) Convex mesh fitting to the NC from (b) used to calculate the volume of NCs. (d) Volume per NC by region calculated using the triple product convex mesh-fitting technique (sample size of NCs given above each box plot). A depiction of brain regions imaged in this work color-coded for comparison with regional box plots (e–g) is presented below. (e) Radius per NC by region assuming them to be spherical. This data agreed with a complementary analysis of the radius by PCF (see Figure S4). (f) Mean number of PSD95 localizations per NC by region. (g) Number of NC/PSD as a percentage by region determined by Pair Correlation Fitting (PCF, mean ± 1 std). (h) Conceptual examples of 1 NC/PSD, 2 NCs/PSD and 3+ NCs/PSD. Visualization was carried out using vLUME desktop. Box plots represent the median and interquartile range, where whiskers extend to the extreme data points which are not considered to be outliers.

A functional advantage of SMLM is its ability to resolve synaptic diversity across increasingly complex spatial scales, beginning with individual proteins, their assembly into NCs, the formation of building blocks within PSDs, and ultimately their differential spatial arrangement across the hippocampus (see Figure 1). Building on our previous genetic labeling strategy,^39^ we imaged intact mouse brain tissue samples of mEos2-labeled PSD95 using DHPSF SR microscopy (see Figure S1 and SI Movie 1). The DHPSF enabled the determination of the true Euclidean coordinates (*x*,*y*,*z*) of each PSD95 molecule with nanometer accuracy. These volumetric pointillist images correspond to the subdiffraction distribution and organization of PSD95 (see SI Movie 2). Like our previous study in 2D^20^ clustering algorithms allowed us to segment the localization data to distinguish thousands of PSDs composed of individual NCs across a large field-of-view in 3D (70 μm × 70 μm × 4 μm, see Figure 2a and Materials and Methods section). Furthermore, our analysis of mEos2 single-molecule photophysics mitigated the risk of molecular overcounting and thus validated our quantitative approach to measuring protein spatial distributions (see Materials and Methods section and Figure S2 for further details).

We first segmented the 3D localization data into NCs within PSDs using DBSCAN (cluster radius of 125 nm and a minimum size of 10 localizations, see Materials and Methods section), thereby distinguishing between NCs within the same PSD and those in adjacent PSDs based on their spatial arrangement and distance. The addition of the *z* dimension better represented the true organization of NCs, and allows us to evaluate not only their radius but also their volume (see Figure 2b,c).

The 3D data can be contrasted with the 2D results in three distinct ways. First, we observe a decrease in the number of PSD95 localizations per cluster from ∼90 (in 2D)^20^ to ∼25 (in 3D). This can be attributed to a ∼65% reduction in sensitivity for the DHPSF method compared to 2D SR microscopy. This combines the ∼30% loss of signal from the inefficiency of the double helix phase mask and reshaping of the PSF footprint into two Gaussian distributions. Second, we observe an increase in NC radii, which is justified by the lower localization precision afforded by the DHPSF compared to 2D, despite improved segmentation in 3D. Finally, we observe an increase in the relative complexity of the PSD subtypes, which can also be attributed to improved 3D segmentation. For example, when observed in 2D, two NCs that are laterally aligned but axially displaced could be misclassified as a single NC per PSD, whereas 3D SR imaging allows for accurate distinction between them.

Analysis of NC populations across the hippocampus (see Figure S3) demonstrated that NC radii, volumes, and the number of localizations per NC were generally uniform across all regions examined, with values of approximately 100 nm, 0.5 × 10^7^ nm^3^, and 25 localizations, respectively (see Figures 2d–f and S4a). Contrastingly, the PSD subtypes of 1, 2, and 3+ NC/PSD varied across the regions. Specifically, the proportion of 2 and 3+ NCs/PSD was typically higher in 3D, except for the DGMO region (see Figures 2g–h and S4b). For example, in the CA1 region, we see the ratio of 1:2:3+ NCs/PSD as 52:18:30, which contrasts with the ratio of 75:19:6 that was observed previously in 2D.^20^ Taken together, these data confirm the presence of NCs in vivo, and their nature as highly conserved building blocks underpinning the structure of PSDs.

### Identification of a “Diffuse” PSD95 Population

**Figure 3:**
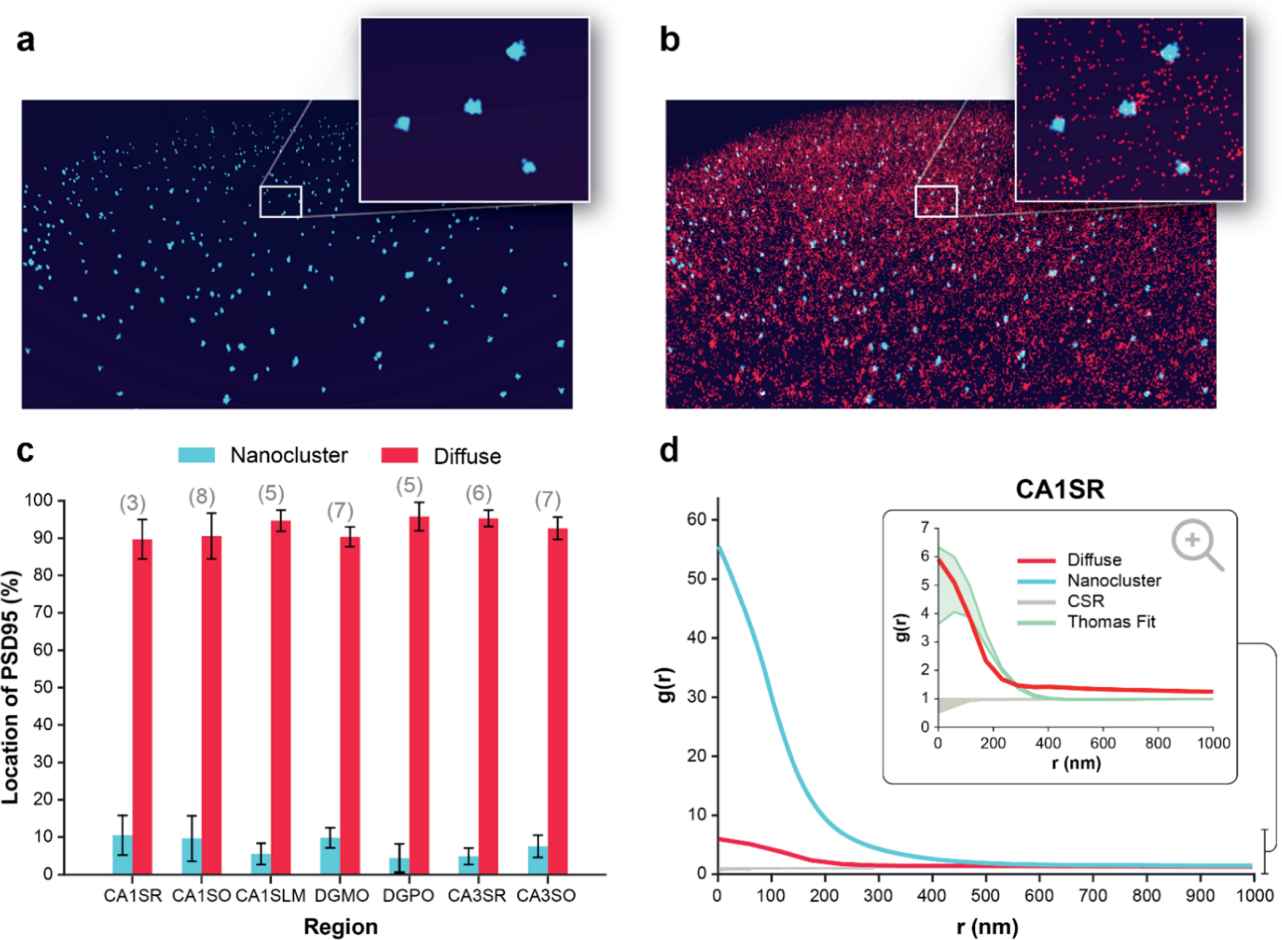
Abundance of PSD95 is found outside of the PSD. (a) Volumetric distribution of PSD95 nanoclusters (blue) in the CA1SR region. (b) Volumetric distribution of NCs and diffuse localizations (red). (c) Distribution of PSD95 localizations associated with the NC and diffuse populations. Error bars represent the standard deviation from the mean (bar) across the replicate tissue sections (shown above each region). (d) PCF analysis of the diffuse (red) and NC (blue) populations in the CA1SR region. This shows the difference in the degree of clustering of the two populations. The two envelopes correspond to the complete spatial randomness (CSR, gray) and Thomas fitting (green) simulations. The red line shows that the diffuse population does not adhere to a complete spatial randomness fit. The magnified region highlights the minor observed clustering present in the diffuse population. PCF analysis results from other regions are presented in Figure S8.

Segmentation-by-clustering allowed us to determine “hot-spots” where localizations were at high density enabling us to distinguish PSDs (see Figure 3a and Materials and Methods section). This approach typically removed randomly distributed localizations, such as noise arising from localization fitting errors, fluorescent impurities on the coverslip, and cutting artifacts to which 2D-SMLM is more sensitive.^40,41^ However, in 3D these interfacial phenomena are not present as we are imaging throughout the entire tissue section and false positive localizations are much less common owing to the complex PSF shape (see Figure S5). The DHPSF therefore allowed us to probe the spatial distribution of all detected PSD95 molecules, not just those segmented in NCs. Upon reevaluating our 3D data, we unexpectedly discovered that most PSD95 are not confined to the PSDs but dispersed as a ‘diffuse cloud’ throughout the brain (see Figure 3b and SI Movie 3). A simple quantification of the number of the clustered and nonclustered localizations revealed that over 90% of protein is in fact not associated with NCs or PSDs, and this effect is broadly uniform over all regions examined (see Figure 3c).

To confirm that this observation was not an artifact of the mEos2-labeled PSD95, we used genetically modified mice expressing eGFP^15^ or the HaloTag^16^ fused to PSD95. Spinning disk confocal microscopy was used to image the eGFP- or Halo-tagged PSD95 in mouse brain sections (see Figure S6). While both methods revealed an abundance of PSD95 outside of the PSDs, the percentage detected using spinning disk confocal microscopy was lower than that observed with mEos2.

To explore this finding in greater detail, we conducted a more sophisticated analysis approach. The localization data was once again segmented into clustered and diffuse data sets, this time using a custom k-Nearest Neighborhood (NN) algorithm (see Materials and Methods section). Subsequently, pair correlation function (PCF) analysis was performed on each data set (see Figure 3d), which represents the probability of encountering a second localization at a given distance relative to a reference point (normalized for the intensity/density of the overall localizations).^42^ For the clustered data, there is a high degree of correlation extending out to distances of ∼400 nm and the cluster radii corresponded well with our previous analysis approach (see Figure S4). On the other hand, PCF analysis of the “diffuse” PSD95 population revealed greater disorder, but spatial correlation was still observed at smaller radii (up to ∼200 nm). For example, the *g*(*r*) values for NCs are ∼7-fold larger than the “diffuse” population in the CA1SR region. To further validate these findings, localization data were simulated using a complete spatial randomness model, in addition to single and double Thomas fitting to generate parameters that were consistent with empirical values (see Figures 3d and S7 and Materials and Methods section). This analysis confirmed that the “diffuse” PSD95 population does not correspond to complete spatial randomness. Instead, it aligns with a Thomas process fitting, indicating a degree of spatial order within the “diffuse” population across all regions (see Figure S8). Taken together, we can conclude that most PSD95 is not localized within the PSD but still retains a small degree of spatial ordering on the nanoscale. We therefore decided to investigate the clustering of diffuse PSD95.

### PSD95 Exists as Multimers within the Diffuse Population

We have recently used single-molecule and super-resolution microscopy to analyze isolated PSD95 supercomplexes from brain homogenate, finding that the majority of PSD95 supercomplexes contain two or more copies of PSD95.^25^ With our knowledge of the existence of the diffuse population here, we next sought to determine whether the individual PSD95 molecules were near each other, and therefore likely to reside as supercomplexes.

To achieve this, we revisited 2D PALM experiments, which gave higher lateral spatial resolution compared to the DHPSF,^36^ and therefore allowed the direct observation of the spatial organization in the diffuse PSD95 subpopulation. The brain sections were immobilized on a glass coverslip and imaged via PALM using total internal reflection fluorescence (TIRF) microscopy (see Materials and Methods section). Each PSD95-mEoS2 molecule was localized with a mean precision of 15.96 ± 2.91 nm (mean ± S.D., *n* = 5546 localizations). The acquired images had a mean resolution of 43.5 nm as determined by Fourier Ring Correlation.^43^ A representative 2D SR image is shown in Figure S9. As with 3D data, clustering was used to distinguish the diffuse population of PSD95 from that residing in NCs. Using this approach, 70% of PSD95 was found in the diffuse population and using DBSCAN (radius of 125 nm, minimum of 10 localizations per cluster). Given that only half of the PSD95 proteins are fused to mEoS2 in heterozygous mice, and that not all mEoS2 will fold correctly, this suggests that the vast majority of PSD95 molecules in the diffuse population are close to a neighboring molecule and are therefore likely to be within the supercomplexes we have previously identified.^25^

**Figure 4:**
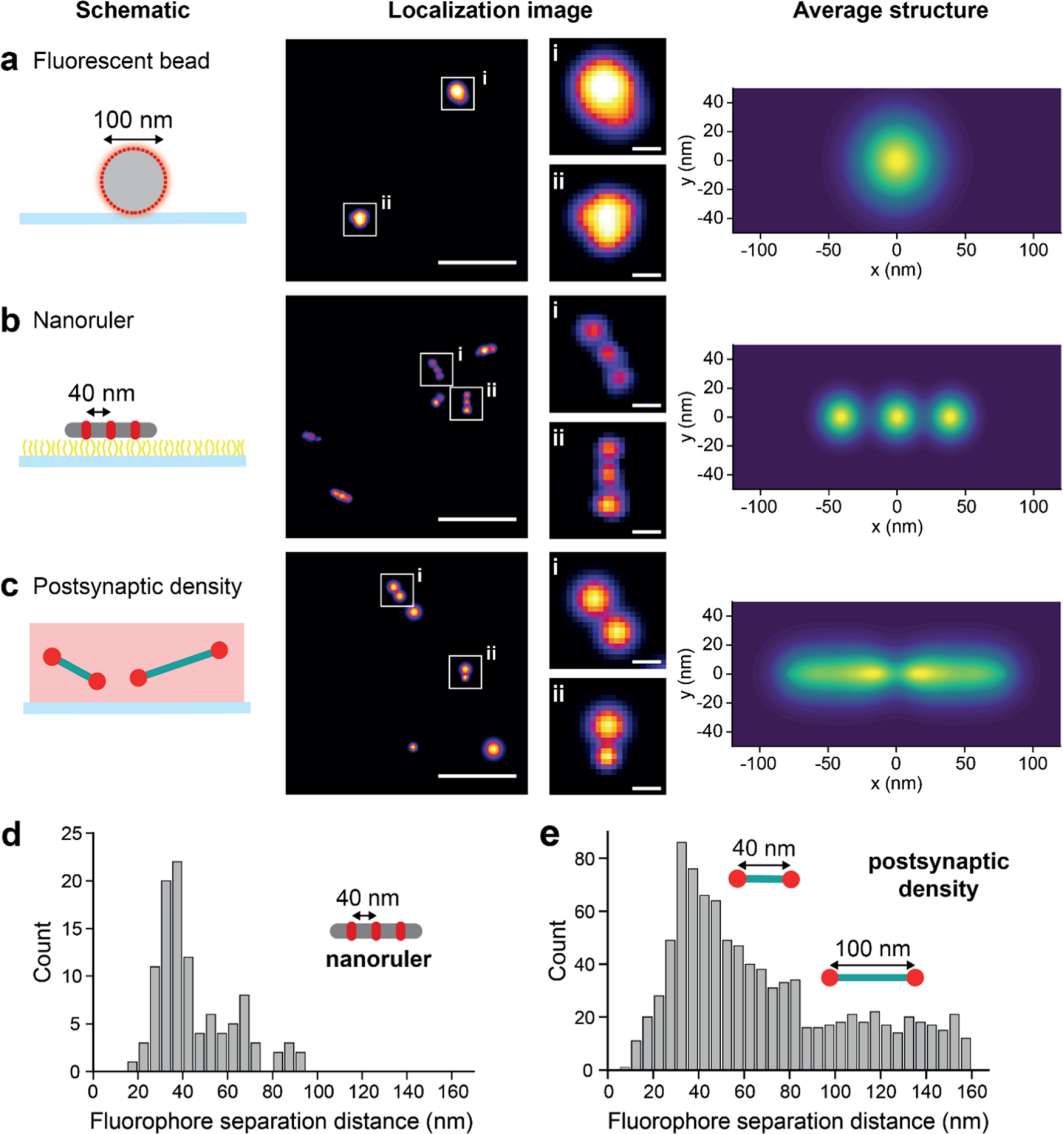
Identification of PSD95 dimers in the diffuse population. (a) SR analysis of TetraSpeck fluorescent beads. An example super-resolution image and the class average of 2534 beads is shown. (b) Super-resolution imaging of DNA-PAINT-based GATTAquant nanorulers. The nanorulers have three fluorophores, each spaced 40 nm apart. The class average of 828 trimeric nanoruler objects is shown. (c) PALM-based imaging of PSD95 endogenously tagged with mEos2 in tissue sections from the CA1 region of the hippocampus. The class average of 1402 dimeric objects is shown. These dimeric objects represent 15% of the total number of clusters present. Histogram of separation distances between fluorophore positions on (d) the GATTAquant DNA nanorulers and (e) the dimeric PSD95 clusters seen in tissue sections. Scale bars are 400 nm (large) and 40 nm (insert).

Image averaging is a proven technique to increase the spatial resolution of SMLM images down to the level of 5 nm or higher.^44^ We validated our image averaging analysis pipeline using TetraSpeck microspheres (see Figure 4a) and GATTAquant PAINT nanorulers (see Figure 4b), which together confirmed our ability to resolve objects separated by 40 nm. Next, we applied class averaging to 1402 supercomplexes containing two PSD95-mEoS2 proteins to investigate their spatial arrangement (see Figure 4c). This revealed that most PSD95 molecules had a peak-to-peak separation of ∼40 nm (see Figure 4d, 1402 dimers). However, a subset displayed a larger separation, resulting in a broad “tail-like” distribution in the class averaged image. Together, Figure 4d and e show the distribution of spatial separations for both nanoruler and PSD95 images side by side. Further quantitative analysis revealed that 72% of PSD95 dimers were separated by a distance of 39 ± 2 nm, and 28% PSD95 dimers were separated by a larger distance of 94 ± 27 nm. The shorter separation distance is close to the spatial resolution that was achievable using PALM, and so we cannot rule out shorter separation distances that we have previously measured using MINFLUX in brain homogenate.^25^ These data are not consistent with higher order geometries, such as trimers and tetramers, from the absence of their characteristic peak patterns of spatial separations (see Figure S10).

Given the existence of dimers, we next validated their spatial dimensions using a complementary labeling strategy. This strategy incorporated a HaloTag conjugated to a bright organic fluorophore to afford higher spatial localization precision for dual-labeled dimeric supercomplexes compared to mEos2. However, a limitation of this labeling strategy was the degree of dual-labeled dimers compared to that achievable with endogenous labeling. Photoactivatable Janelia Fluor 549 was selected for these experiments, which also revealed the existence of two spatially separated PSD95 populations at ∼40 and ∼100 nm, in agreement with our mEos2-PALM experiments (see Figure S11).

## Discussion

We demonstrate, to our knowledge, the first implementation of DHPSF SR microscopy in tissue sections, showing that the technique can super-resolve single fluorescent proteins in 3D, deep within brain tissue. Use of the DHPSF enabled us to enhance our understanding of the spatial organization of PSD95 in brain tissue. This approach confirms the presence of NCs of PSD95 in the PSD of excitatory synapses previously observed using 2D SMLM. Although the PSDs are the most obvious structures in the tissue sections, an abundance of PSD95 is found in diffuse clouds outside of these, most likely within the dendritic tree. This population is observed in both the CA1 and DG, which are populated by pyramidal neurons and granule cell neurons, respectively, suggesting that the diffuse population is found in the major classes of excitatory neurons and is not neuron-type-specific.

To ensure that the diffuse population was not an artifact of the mEos2-labeled PSD95, we imaged genetically modified mice expressing PSD95 fused to either eGFP or HaloTag using spinning disk confocal microscopy. Both imaging approaches confirmed the presence of PSD95 outside the PSDs; however, the proportion of diffuse PSD95 was lower than that observed with mEos2 labeling. This difference could be attributed to the lower detection efficiency of spinning disk confocal microscopy, or the poorer fitting of double helices in high-density/high-background PSD regions, which may exclude some PSD95 molecules. The finding that the majority of protein resides outside of the PSD can be rationalized by considering that the total volume of PSDs is minuscule: a simple back-of-the-envelope calculation estimates that the total volume of all PSDs is ∼0.02% of the entire mouse brain (see Section S1). While we cannot entirely rule out the possibility that a minority of these localizations may have been misidentified and belong to NCs within PSDs, their spatial uniformity throughout the imaging volume strongly suggests that the majority are not associated with PSDs. Additionally, there is no observed increase in density of these signals around the PSDs, further supporting this conclusion.

Further examination of the diffuse population identified a lesser degree of clustering, which we subsequently assigned as PSD95-containing supercomplexes, the majority of which contain two units. This finding supports biochemical studies on those purified from the mouse brain homogenate.^13,26^ We have also recently used single-molecule and super-resolution microscopy to demonstrate that the majority of supercomplexes isolated in mouse brain homogenate contain two units of PSD95.^25^ Furthermore, we showed that in addition to the entire supercomplex being turned over, protein can be degraded and replaced within each supercomplex, a finding that could have ramifications for long-term memory storage by proteins that have a lifetime ranging from hours to months.^16^ In this study, we identified the presence of supercomplexes outside the PSD, although whether protein exchange occurs in this location remains unclear.

We also showed that the diffusely distributed supercomplexes containing two units form two populations with a spatial separation of 39 ± 2 or 94 ± 27 nm, which may be indicative of a differential composition of interacting proteins. Although we could not resolve supercomplexes within the densely packed NCs in the PSD, biochemical studies indicate that they also comprise two PSD95 units, leading us to speculate that PSD95 forms complexes in dendrites that are transported into synapses where they pack together into the NCs of the PSD.

Our results indicate that while the dense packing of complexes within the PSD enables PSD95 to be visible by confocal microscopy, the single molecule sensitivity of the DHPSF enabled the much larger diffuse population to be resolved and quantified and not manifested as low level autofluorescence. Therefore, the application of DHPSF and other multidimensional SR microscopy methods could further our understanding of whether other synaptic proteins are more widely distributed than previously known.

Mice carrying genetically tagged PSD95 have enabled the most comprehensive analysis of the spatial organization and distribution of any synaptic protein to date, spanning scales from single molecules to the whole brain.^16,25,45−47^ These studies have revealed how PSD95 undergoes a hierarchical assembly of increasing molecular complexity from proteins, complexes, supercomplexes, and nanoclusters to form the mature and functional postsynaptic terminal of excitatory synapses. Moreover, the differential distribution of these supramolecular structures into different synapses on the dendritic tree, and between neuron types, produces a remarkable spatial diversity of synapses described by the synaptome architecture of the brain.^15,17^ The stability of the synaptome architecture of PSD95-expressing synapses in the adult brain, despite PSD95 turnover, suggests that the regulation of the synaptic and extrasynaptic protein pools, as described in this study, is tightly regulated.^16,17^ Our approaches, which are applicable to any synaptic protein, lay the groundwork for exploring the dynamics of PSD95 supercomplex populations, their mechanisms of transport, and assembly into synapses.

## Materials and Methods

### Tissue Sectioning

Animal procedures were performed in accordance with U.K. Home Office regulations and approved by Edinburgh University Director of Biological Services. Generation and characterization of PSD95-mEos2 fluorescent knock-in mouse line was described previously.^20^ Adult 2- to 3-month-old heterozygous PSD95-mEos2 (*n* = 3) and wild-type C57BL/6 (*n* = 1) mice were fully anesthetized by intraperitoneally injecting pentobarbital (0.10 mL, Euthatal) and, following the opening of the thorax, were perfused with PBS (1 × 12 mL) and postfixed with 4% paraformaldehyde (PFA; #043368.9M, Thermo Scientific). The brains were dissected and placed in 4% PFA and incubated at 4 °C for 3–4 h. 4% PFA solution was replaced with 30% sucrose solution and the brains were left at 4 °C for 48–72 h. Brain tissue was embedded in OCT embedding matrix (CellPath) inside a plastic mold (#1003684132, Sigma-Aldrich) and frozen with liquid nitrogen and isopentane. Brains were stored at −80 °C for up to 1 month. Glass coverslips (#1; 631–0142, VWR) were plasma cleaned and coated with poly-d-lysine (PDL, 1 mL, 0.22 μm filtered) for 30 min. Coverslips were then washed with distilled water (dH_2_O). Fluorescent nanodiamonds (0.1 μm, 50 μL, nitrogen vacancy >900 NV/particle, Sigma-Aldrich) were applied and incubated for 30 min. The coverslips were then washed with dH_2_O. Frozen brain samples were cut at 6 μm thickness using a cryostat (NX70 Thermo Fisher) to obtain coronal brain sections referring bregma level between −1.94 and −2.46 mm, capturing dorsal hippocampus. Cut brain sections were placed on the coverslips. A drop of dH_2_O was placed on a glass slide prior to picking up the brain sections to ensure the brain tissue laid flat. After cutting, brain sections were left to dry in the dark at room temperature overnight and were then stored at −80 °C for up to 1 month.

### Double Helix Point Spread Function (DHPSF) Microscopy

The DHPSF is a custom widefield microscopy platform that incorporates additional optical elements to transform the shape of the standard PSF to encode the axial position of single emitters. This was built incorporating a 1.27 NA water-immersion objective lens (Plan Apo VC 60×, Nikon, Tokyo, Japan) to image above the coverslip surface. The DHPSF transformation was achieved by introducing additional optics into the emission path of a conventional fluorescence microscope (Eclipse Ti–U, Nikon) with the objective lens mounted onto a scanning piezo stage (P726 PIFOC, PI, Karlsruhe, Germany). A 4f system of lenses placed at the conjugate image plane relayed the image onto an EMCCD detector (Evolve 512 Delta, Photometrics, Tucson, AZ; 16 μm pixel size). A double-helix phasemask (PM) (DoubleHelix, Boulder, CO; 580 nm optimized) placed in the Fourier plane of the 4f system performed the DHPSF transformation. Excitation and activation illumination was provided by 561 nm (200 mW, CoboltJive 100, Cobolt, Solna, Sweden; ∼0.25 kW cm^–2^) and 405 nm (120 mW, iBeam smart-405-s, Toptica, Munich, Germany; <7 W cm^–2^) lasers, respectively. The lasers were circularly polarized, collimated, and focused to the back aperture of the objective lens. The fluorescence signal was then separated from the excitation beams into the emission path by a quad-band dichroic mirror (Di01-R405/488/561/635–25 × 36, Semrock, Rochester, NY) before being focused at the image plane by a tube lens (*f* = 200 mm, Nikon). Finally, long-pass and band-pass filters (BLP02–561R-25 and FF01–580/14–25, Semrock) placed immediately before the camera isolated the fluorescence emission. An exposure time of 50 ms was used to image the PSD95-mEos2.

The conversion gain of the EMCCD detector (Evolve 512 Delta, Photometrics) for DHPSF microscopy was measured frequently using a custom MATLAB script (https://github.com/TheLeeLab/cameraCalibrationCMOS). The in-built “Rapid-Cal” function also served to calibrate the EM gain register on a weekly basis. The CCD gain value was measured at 0.154 counts/photoelectron which—when operated at an EM gain of 250—gave a total conversion gain value of 38.5 counts/photoelectron.

### Preparation of Fluorescent Bead Samples for DHPSF Calibration

Glass slides (VWR, 631–1570) were cleaned under argon plasma (PDC-002, Harrick Plasma, Ithaca, NY) for 1 h and incubated with poly-l-lysine (PLL, 50 μL, 0.1% w/v, Sigma-Aldrich, P820) for 30 min. Glass slides were then washed with PBS (3 × 50 μL) and incubated with fluorescent beads (50 μL, 4.6 × 10^7^ particles mL^–1^; 0.10 μm, TetraSpeck microspheres, Thermo Fisher, Waltham, MA, USA) for 2 min before washing further with PBS (3 × 50 μL). The sample was then transferred to the microscope for imaging. Here, the piezo-mounted objective was scanned axially through the sample in 60 nm steps over 4 μm, recording 10 frames of 30 ms exposure at each step. This calibration file was subsequently used for 3D reconstruction using easy-DHPSF fitting software.^48^

### DHPSF Localization Analysis

Initially, ImageJ was used to remove autofluorescence background from raw localization data sets by subtracting the average intensity offset of the *z*-stack. The immobile fiducial marker was therefore processed separately since it was steadily fluorescent. Superlocalization of the DHPSF was conducted using easy-DHPSF, which has been described previously.^48^ In brief, single-molecule fluorescence was identified using template matching and localized in 3D by double Gaussian fitting of the DHPSF using nonlinear least-squares minimization. The lateral position of each fluorophore was determined from the midpoint of both Gaussian lobes, while the *z* position was calculated from the angle between the two Gaussian lobes using the calibration curve produced from fluorescent beads. The fitting precision of the microscope platform has been evaluated previously at ref (49) (Figure 1b therein) using eq 1
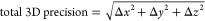
1where Δ*x*, Δ*y*, and Δ*z* refer to the standard deviation in the position of a point emitter at a given photon flux. This gave a total 3D precision of 38 ± 8 nm for mEos2 (see Figure S1).

To avoid overcounting errors the inherent photophysical properties of mEos2 were evaluated for DHPSF microscopy. Prior to cluster analysis, the proportion of repeated blinks from the same fluorophore was determined experimentally. The results are presented in Figure S1, which show that the probability of detecting a recurring signal from the same fluorophore within a sphere of 50 nm radius is 2% over a 50-frame sliding window. This is lower than typically observed for mEos^50^ but can be rationalized by to the low photon-efficiency of the DHPSF phase mask, which reduces overall signal by approximately 20–30%,^51^ and the higher laser powers used in this work. Therefore, the risk of overcounting molecules was considered negligible.

### Fiducial Correction for DHPSF Microscopy

Drift correction was performed by localizing the position of a fiducial marker in each frame and subtracting the resulting 3D points from all localizations in the corresponding frame. Fluorescent nanodiamonds (10 μg mL^–1^ in dH_2_O; 100 nm, nitrogen vacancy >900 NV particle^–1^, Sigma-Aldrich) were used as fiducial markers to account for 3D focal drift. Microscope slides were incubated with PLL (50 μL, 0.1% w/v, Sigma-Aldrich, P820) for 30 min and washed with PBS (3 × 50 μL) before adding the nanodiamond solution. The sectioned brain was then adhered to the coated slides and the nanodiamonds left to diffuse through the sectioned tissue to reside at different depths.

### Nanocluster Analysis

The size, volume and number of localizations per cluster were calculated and visualized using vLUME desktop software (see Figure 2e–g).^52^ First, the NC and diffuse populations were segmented using DBSCAN (radius = 125 nm, *N*_localizations_ ≥ 10). Radius and volume were determined using bespoke analysis scripts in vLUME that implemented triple product convex mesh fitting to calculate the volume of a parallelepiped (composed of six equal tetrahedra). In brief, the cross product was used to find the area of the parallelogram formed by two vectors. The volume was then obtained by projecting the diagonal length onto the base’s normal (which is implicitly done via the dot product) as the cross-product’s area vector served as the scaled normal. The resulting volume was either convex or concave, and was approximated by measuring a spherical volume with a radius equal to the distance from the centroid to the furthest vertex.

### Pair Correlation Function Analysis

First, the nanocluster (NC) and diffuse populations were segmented using a mixture model applied to the *k*-nearest neighbor (nn = 10) distance computed for each point (as implemented by the *nnclean* function in the R *spatstat* library).

Pair Correlation Function (PCF) analysis was then implemented to determine the extent of clustering for the NC, PSD and NC/PSD distributions using three fitting algorithms.^53^ The first algorithm comprised a complete spatial randomness test (CSR, see Figure S6a) for which simulated data sets of random points were generated with equal volume and localization density as the raw data (*N* = 49). The second was a single Thomas fit (Neyman-Scott cluster process,^54^ where the clusters follow 3D Gaussian distributions around parent points, see Figure S6b). This process generated simulated data sets of clustered localizations where parameters were informed by experimental data (125 nm radius, 10–50 localizations per cluster, *N* = 49). Finally, a double Thomas fitting algorithm produced simulated cluster-of-clusters data sets consistent with the experimentally observed NC population only (see Figure S2c). For each population, a model selection and a parameter estimation step were applied.

Both model selection and parameter estimation were based on the PCF used, a summary statistic with a known analytical expression for the three models above. A Poisson process (describing CSR) has a *g*(*r*)=1. A modified Thomas process has a *g*_1_(*r*) described by eq 2
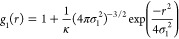
2and a double Thomas process has a *g*_2_(*r*) described by eq 3
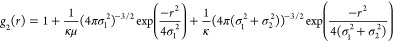
3where κ is parent intensity.

Fitting the experimental PCF to the theoretical description allowed for estimation of the model parameters. For the Thomas process this included cluster size (σ) and cardinality (μ). For double Thomas process this includes the cluster sizes (σ_1_ and σ_2_) and cardinalities (μ and ν). In order to select the best model, envelope tests were performed (*N* = 49 replicates) and the envelope of the PCF summary statistics were computed. The empirical PCF was superimposed on the envelope graphs and the overlap indicates how well the model describes the experimental data.

In summary, the NC populations were fitted to Thomas and double Thomas processes, while the diffuse populations were fitted to CSR and Thomas processes. Representative code and data sets are available via the Zenodo repository associated with this work.^53^

### 2D PALM Imaging

Two-dimensional PALM experiments were performed on a home-built TIRF microscope described previously.^55^ Briefly, collimated laser light at 405 nm (Cobolt MLD 405–250 Diode Laser System, Cobalt, Sweden) and 561 nm (Cobolt DPL561–100 DPSS Laser System, Cobalt, Sweden) was aligned and directed parallel to the optical axis at the edge of a 1.49 NA TIRF objective (CFI Apochromat TIRF 60 × C Oil), mounted on an inverted microscope (Ti2, Nikon). The microscope was fitted with a perfect focus system to autocorrect the z-stage drift. Fluorescence collected by the same objective was separated from the returning TIR beam by a dichroic mirror (Di01-R405/488/561/635, Semrock, Rochester, NY), and was passed through appropriate emission filters (LP02–568RS, FF01–587/35, Semrock, Rochester, NY). Fluorescence was then passed through a 2.5× beam expander and recorded on an EMCCD (Evolve 512 Delta, Photometrics, Tucson, AZ; 16 μm pixel size) operating in frame transfer mode. This produced a pixel size at the sample plane of 103 nm. The CCD gain was measured at 0.0870 counts/photoelectron which, when operated with an EM gain of 250, gave a total conversion gain of 21.0 counts/photoelectron. Images were recorded with an exposure time of 50 ms. The microscope was automated using the open-source microscopy platform Micromanager.^56^ Borosilicate glass coverslips (20 mm × 20 mm, VWR International) were cleaned under argon plasma (Zepto, Diener) for 30 min to remove any fluorescent residues. The tissue sections, prepared as above, were then added to the coverslips. The samples were illuminated with 15 cycles at 405 nm (75 W cm^–2^, 1 s) and 561 nm (1.5 kW cm^–2^, 10 s) irradiation until all molecules were photobleached. For the identification of PSD95 dimers in the diffuse population, GATTA-PAINT 40 nm nanorulers (PAINT 40R, fluorophore: ATTO 655) and TetraSpeck microspheres (100 nm, T7279) were used.

The data was analyzed using the Peak Fit (GDSC SMLM, ImageJ plug-in) to output localizations of the individual fluorophores. A signal strength threshold of 20 and precision threshold of 40 nm were used. Focal drift was corrected for using the in-build Drift Calculator function in the GDSC SMLM plugin. A custom written MATLAB clustering script was used to classify localizations into clusters. All localizations were first ordered by precision from low to high. To correct for multiple localizations of the same fluorophore, the script combined localizations within the precision of another localization into one object. The distances between all objects were then calculated. Objects separated by less than 160 nm were grouped into clusters. The number of objects in each cluster were then counted. Clusters containing one object were defined as “monomeric”, clusters containing two objects were defined as “dimeric” and so forth. Information pertaining to the clusters (number of objects, *x*–*y* position of objects, average precision) was output as a text file.

### Class Averaging of 2D Data

To produce the class averaged images, the center point of each feature (PSD95 dimers, DNA nanorulers, or fluorescent beads) was found and the object rotated into the *y* = 0 plane. The average of all objects was then calculated and plotted as a surface plot, with the *z* axis representing the density of features in the *x*–*y* plane. The average precision of the localizations used in the class average was 25 ± 5 nm, which was determined from the photon budget of mEos2, and subsequently photon-matched for the beads and nanorulers.

### Preparation of Histological Sections

Mice were anesthetized by intraperitoneal injection of dolethal (pentobarbital). The animal was then transcardially perfused with PBS (10 mL) followed by paraformaldehyde (PFA, 10 mL, 4% in PBS). The brain was dissected, kept in 4% PFA for 3 h, and then transferred to 30% sucrose in PBS. After 48–72 h of incubation at 4 °C, the sample was embedded in OCT and frozen using liquid nitrogen. The samples were kept at −80 °C until use. Frozen brains were cut at 18 mm thickness using a cryostat (CM3050 S, Leica) to obtain sagittal sections. The brain sections were placed on Superfrost Plus glass slides (Epredia) and left to dry up in the dark at room temperature overnight and kept at −20 °C until use. As for PSD95-eGFP sections, the samples were incubated in PBS for 15 min and then embedded in MOWIOL solution with coverslips.

### Labeling Brain Sections with HaloTag Ligand

PSD95-Halo brain sections were first incubated in PBS for 10 min and then in PBS with/without JF552 HaloTag ligand (800 nM). The sections were washed 3 times with PBS containing 0.2% Triton X-100 and then twice with PBS without detergent. The labeled sections were embedded in MOWIOL solution with coverslips.

### Confocal Microscopy

Imaging was performed using spinning disk microscope (Nikon, Eclipse Ti2) equipped with a 100× objective lens. Images of 862 × 826 pixels in size and 16-bit depth were obtained. eGFP and JF552 were excited at 488 and 561 nm laser, respectively.

## Data Availability

Supplementary analysis code and raw data sets are available at: https://zenodo.org/records/13332752.^53^

## Supporting Information Available

Estimation of PSD volume to total brain volume; distribution of single-molecule intensities; effect of repeat photo-blinking from a single FP on analysis; negative control of WT brain tissue, and pair correlation fit analysis schematic (PDF)
PSD95-mEos2: Raw DHPSF localization data (Movie 1); PSD95-mEos2: Diffraction-limited vs super-resolved (Movie 2), and PSD95-mEos2: Clusters and supercomplexes (Movie 3) (ZIP)

## Notes

**Statistics and Reproducibility** No statistical methods were used to predetermine sample size. Where relevant, sample sizes are reported in the figure legends, and all experiments were performed independently at least three times to ensure results were repeatable.
