# Supplementary material for: 3D Super-Resolution Imaging of PSD95 Reveals an Abundance of Diffuse Protein Supercomplexes in the Mouse Brain
Source: ACS Chem Neurosci. 2024 Dec 20;16(1):40–51. doi: 10.1021/acschemneuro.4c00684 (PMC11697326; doi:10.1021/acschemneuro.4c00684)
Supplement: Supplementary file 1 — cn4c00684_si_001.pdf [file cn4c00684_si_001.pdf]

Supplementary Information

3D super-resolution imaging of PSD95 reveals an abundance of diffuse protein supercomplexes in the mouse brain

Sam Daly[a1], Edita Bulovaite[b,1], Anoushka Handa[a], Katie Morris[c,d], Leila Muresan[e], Candace Adams[c,d], Takeshi Kaizuka[c,d], Alexandre Kitching[g], Alexander Spark[g], Gregory Chant[a], Kevin O'Holleran[e,f], Seth G. N. Grant[b,2], Mathew H. Horrocks[c,d,2], and Steven F. Lee[a,2].

[a] Yusuf Hamied Department of Chemistry, University of Cambridge, Cambridge, CB2 1EW, UK.
[b] Genes to Cognition Program, Centre for Clinical Brain Sciences, University of Edinburgh, Edinburgh, EH16 4SB, UK.
[c] IRR Chemistry Hub, Institute for Regeneration and Repair, University of Edinburgh, Edinburgh, EH16 4UU, UK.
[d] EaStCHEM School of Chemistry, University of Edinburgh, Edinburgh, EH9 3FJ, UK.
[e] Cambridge Advanced Imaging Centre, University of Cambridge, Cambridge, CB2 3DY, UK.
[f] ZOMP, Maxwell Centre, JJ Thomson Avenue, Cambridge, CB3 0HE, UK.
[g] Lume VR Ltd. 26 Stanley Road, Oxford, OX4 1QZ, UK.

[1]S.D. and E.B. contributed equally to this work.
[2]To whom correspondence may be addressed.

e-mail: seth.grant@ed.ac.uk, mathew.horrocks@ed.ac.uk, or sl591@cam.ac.uk.

Table of contents

S1. Estimation of PSD volume to total brain volume S3

S2. Supplementary figures S4

S3. Description of supporting movies S13

References S14

S1: Estimation of PSD volume to total brain volume

The average volume of the mouse brain is 415 mm^3, and the volume of each brain region is given in **Table S1**.[1] Data describing the density of excitatory synapses in each region was taken from Santuy *et al.* and is shown in the second column of Table S1.[2] The number of excitatory synapses in each region was calculated, along with the total number of excitatory synapses.

The volume of the PSD was calculated assuming the PSD is an oblate spheroid, with the area of the circular 2D projection being 0.12 µm^2,[3] and the thickness being 23 nm.[4] Using Equation S1 where A is the area of the circular 2D projection and r is the semi-minor axis of the oblate spheroid, the volume of the PSD was calculated to be 1.4×10^{-21} m^3.

$$V = \frac{1}{3}\pi r \times A \tag{S1}$$

The total number of excitatory synapses in the mouse brain was calculated to be 4.4×10^{11}.

The total volume of PSDs in excitatory synapses was calculated to be 6.2×10^{-11} m^3.

The volume of the mouse brain is 4.15×10^{-7} m^3.

The volume of PSDs is therefore only 0.02% of the total brain volume.

Table S1. Information used in the estimation of PSD volume to total brain volume.

**Region**	**Volume (x10^{10} µm^3)[1]**	**Number of synapses per unit volume (µm^{-3})[2]**	**Total synapses ($\times 10^{10}$)**	**Total volume of PSDs ($\times 10^7$ µm^3)**
Isocortex	11	1.7	19	27
Hippocampus	2.3	1.8	4.1	5.7
Striatum	1.5	1.3	2.0	2.8
Diencephalon	4.0	0.79	3.2	4.5
Brainstem	5.0	0.26	1.3	1.8
Cerebellum	5.2	0.47	2.4	3.4
Fiber tracts	1.4	0.020	0.028	0.039
Other	11	1.1	12	17
**Total**	**41**	**-**	**44**	**62**

S2: Supplementary Figures

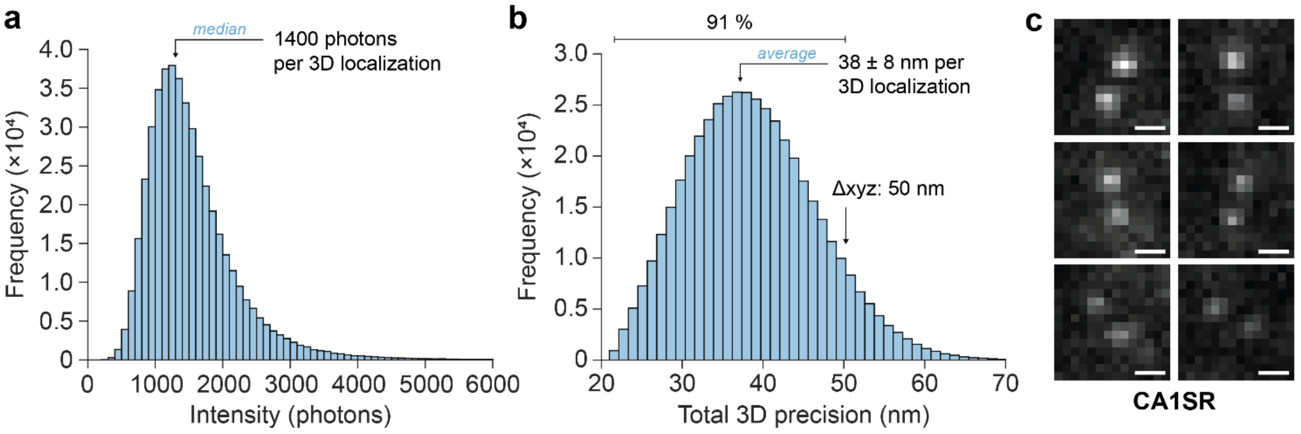

Figure S2. Distribution of single-molecule intensities. a Distribution of PSD95-mEos2 single-molecule intensities via DHPSF SR microscopy. A median of 1400 photons were detected per molecule in 3D in CA1SR region (representative of all regions) using easy-DHPSF software. **b** Empirically determined localisation precision using data from Ref. 5 (Figure 1b, same microscope). An average 3D precision of 38 ± 8 nm was determined. 91% of localizations were below 50 nm precision. **c** Raw DHPSF single molecule localisation data illustrating the range of signal-to-noise ratio obtained.

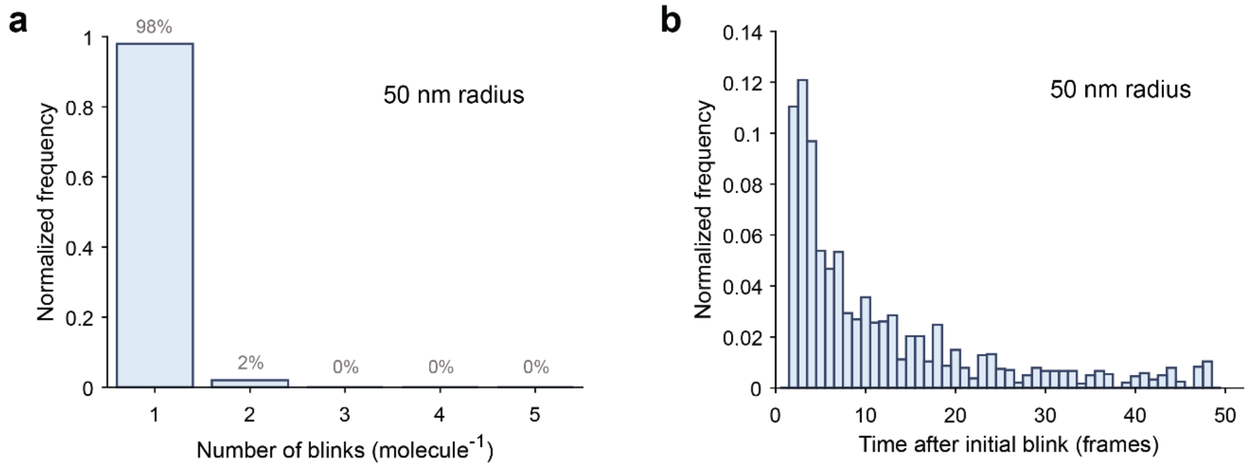

Figure S1. Effect of repeat photo-blinking from a single FP on analysis. a The optimisation of frame rate, illumination power density and photon efficiency of the DHPSF with mEos2 meant that 98% of all fluorescent events were captured within a single frame. **b** The 2% of molecules that blinked twice were analysed temporally within a 50-frame window. For this subset of mEos2 molecules, a second blink was detected from 51% of molecules within 8 frames following the initial blink. A radius of 50 nm was selected based on the precision histogram in **Figure S1b**.

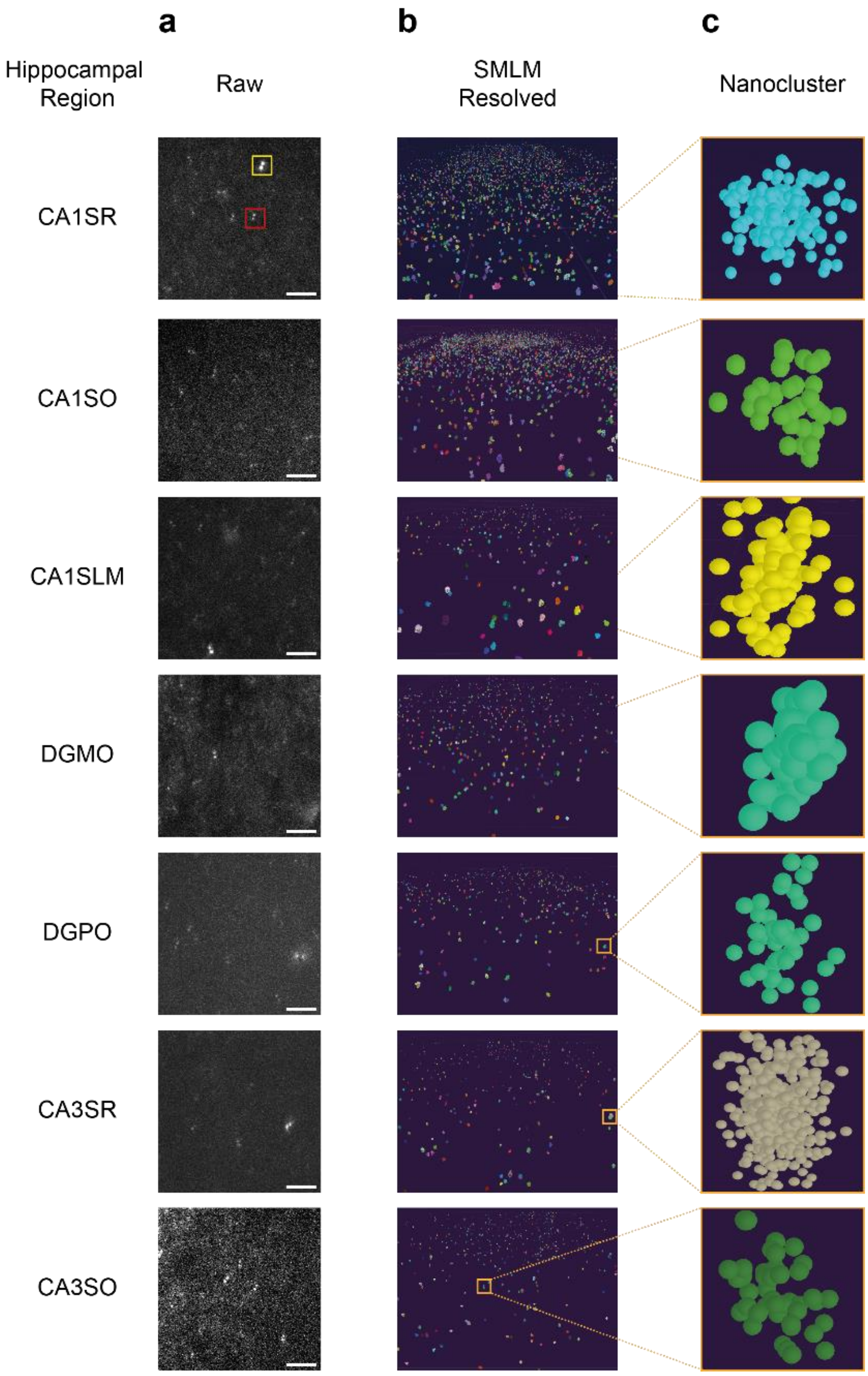

Figure S3. Example DHPSF data collected from different brain regions. Seven examples of collected data, with super-localised reconstructions and an example of a single nanocluster. **a** The seven hippocampal regions studied in this paper (CA1SR, CA1SO, CA1SML, DGMO, DGPO, CA3SR, CA3SO) and a representative frame of raw DHPSF data from each of these regions. Highlighted in the first box (CA1SR) are the fiducial marker (yellow) and a single PSD95-mEos2 localisation (red). Scale bar is 10 µm. **b** The corresponding super-resolved data visualised in vLUME from 50,000 frames in each hippocampal region. Each dataset shows the PSD95 nanoclusters imaged in the hippocampus represented as demarcated colours. Each region is approximately 100 × 100 × 4 µm. **c** Example of a single PSD95 nanocluster (maximum radius of 125 nm) in each hippocampal region. CA1SR, CA1 stratum radiatum; CA1SO, CA1 stratum oriens; CA1SLM, CA1 stratum lacunosum-moleculare; DGMO, dentate gyrus molecular layer; DGPO, dentate gyrus polymorph layer; CA3SR, CA3 stratum radiatum; CA3SO, CA3 stratum oriens.

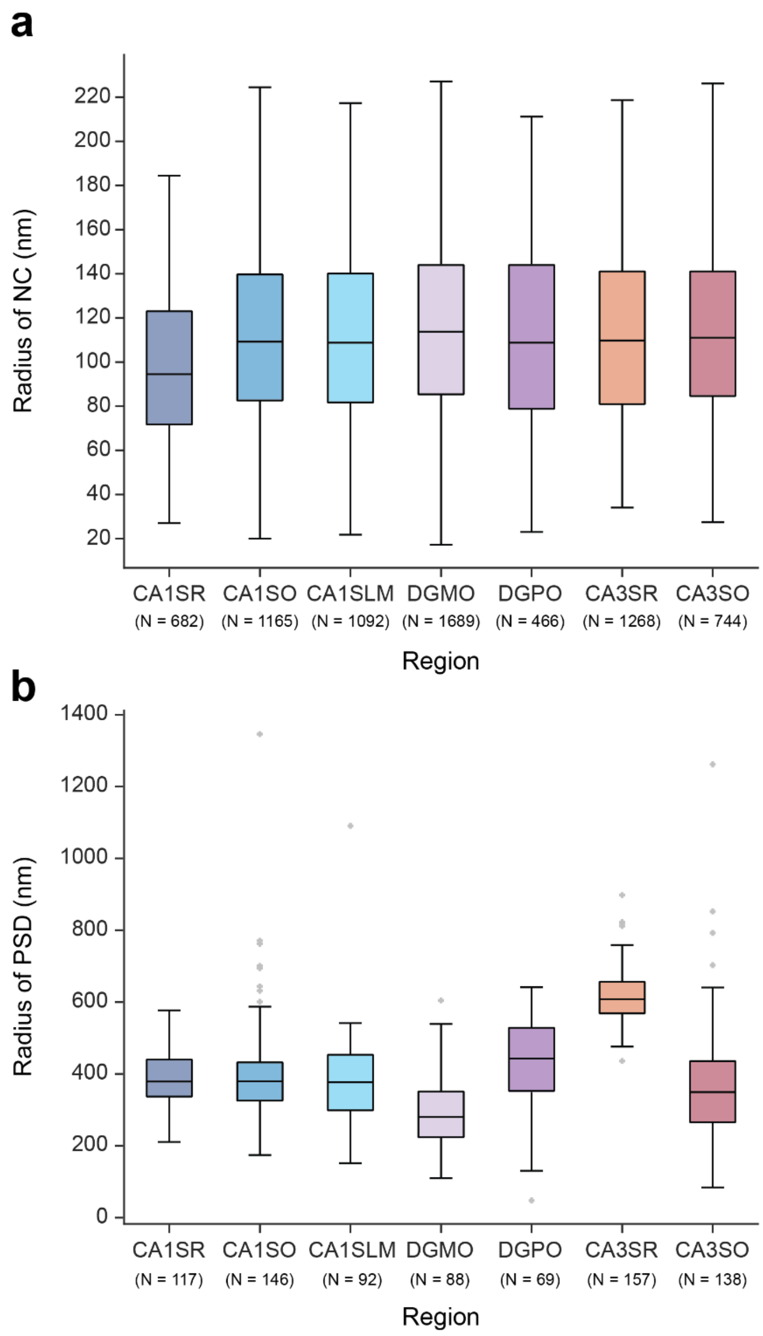

Figure S4. Radii of NCs and PSDs determined using PCF analysis. a Nanocluster radius by region calculated by Single Thomas Pair Correlation Fit. **b** Postsynaptic density radius calculated by Double Thomas Pair Correlation Fit. Box plots represent the median value and interquartile range, where whiskers denote the lower and upper limits not considered outliers. Outliers are represented by grey points. Number of NCs/PSDs analyzed is denoted by N for each box plot.

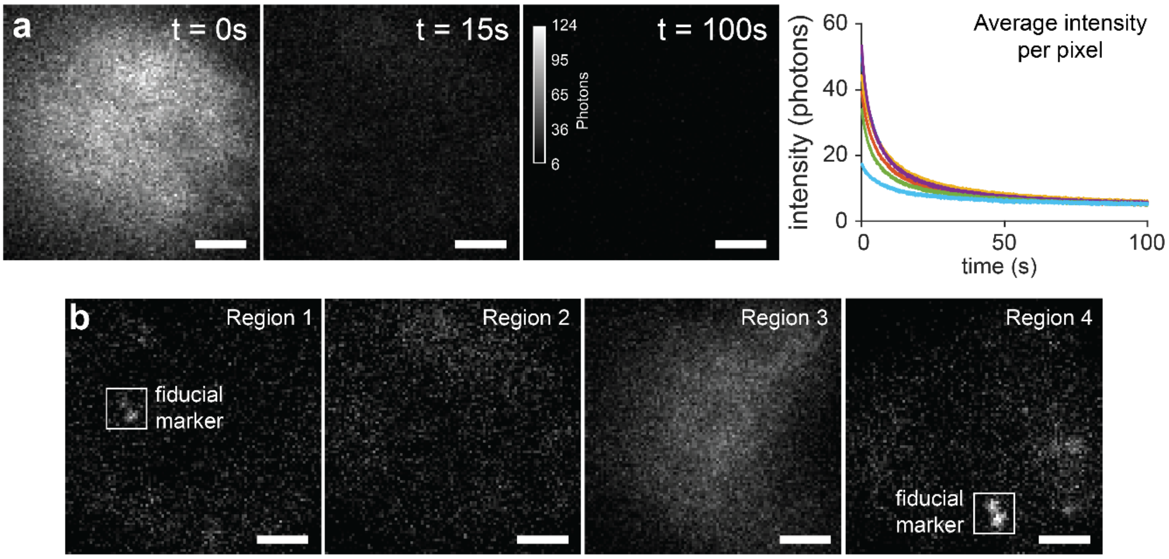

Figure S5. Negative control of WT brain tissue. a Representative images of wild-type (WT, no mEos2-PSD95) brain tissue used as a negative control under identical imaging conditions to experimental samples (see **Methods**). This control confirms that background fluorescence was almost completely bleached after 15 s (300 frames, 50 ms exposure) of illumination. During data acquisition in this work, the sample was first bleached for this duration. Scale bar is 5 µm. A plot of average pixel intensity over a 100 s (2000 frame) period. Colours represent repeats in different regions of the hippocampus (N = 6). **b** No false positive localisations were detected in the WT brain tissue as shown by the representative frames. Fluorescent nanodiamonds (FNDs) used as fiducial markers are indicated with white boxes, which are expanded on the right as a z-projection over 2000 frames (100 s) to confirm their constant emission.

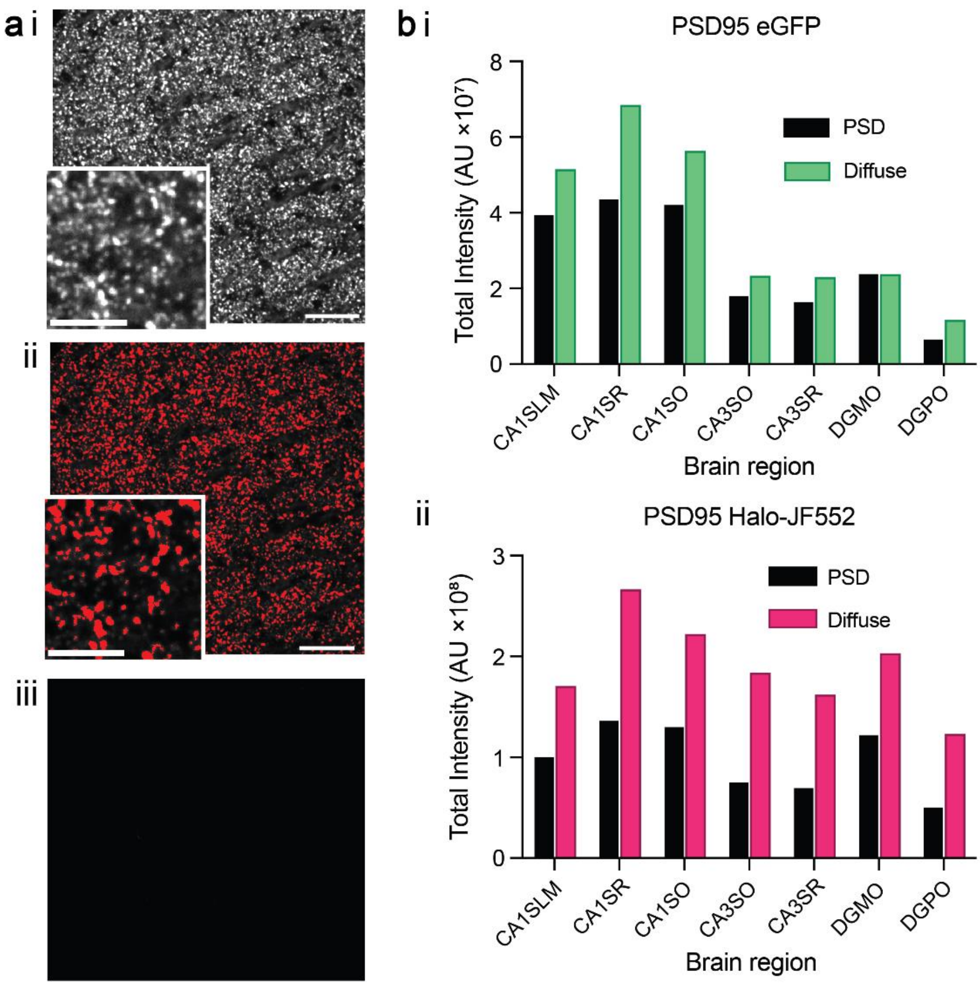

Figure S6. Quantification of PSD95 found in the PSD or as diffuse protein using spinning disk confocal microscopy. ai Representative image of hippocampal sub region (CA1SR) of a PSD95-Halo mouse (Ref. 6) labeled with a JaneliaFluor-552 (JF552) HaloTag ligand and imaged using spinning disk confocal microscopy. Scale bar 100 µm, inset scale bar 5 µm. **aii** Same field of view showing segmentation of PSDs. PSDs are defined by applying a threshold using Otsu's method. Intensities above the threshold were classified as PSDs, while diffuse regions were classified as areas below the threshold. **aiii** Hippocampal sub-region (CA1SR) of an unlabeled PSD95-Halo mouse (contrast-matched to **ai**). **b** Total intensities from PSDs and diffuse regions across different hippocampal subregions taken from an **i** PSD95-eGFP mouse (Ref. 3), and **ii** PSD95-Halo mouse labeled with a JF552 HaloTag ligand. The total intensities for PSD (Black) and diffuse (Green eGFP/Magenta JF552) regions were background-subtracted based on the average of corresponding unlabelled brain regions. This analysis highlights the dominant contribution to the total intensity from a diffuse population of PSD95 outside of the PSD as well as the variation of populations across hippocampal regions. Homozygote of PSD95-Halo and heterozygote of PSD95-eGFP were used for the experiment.

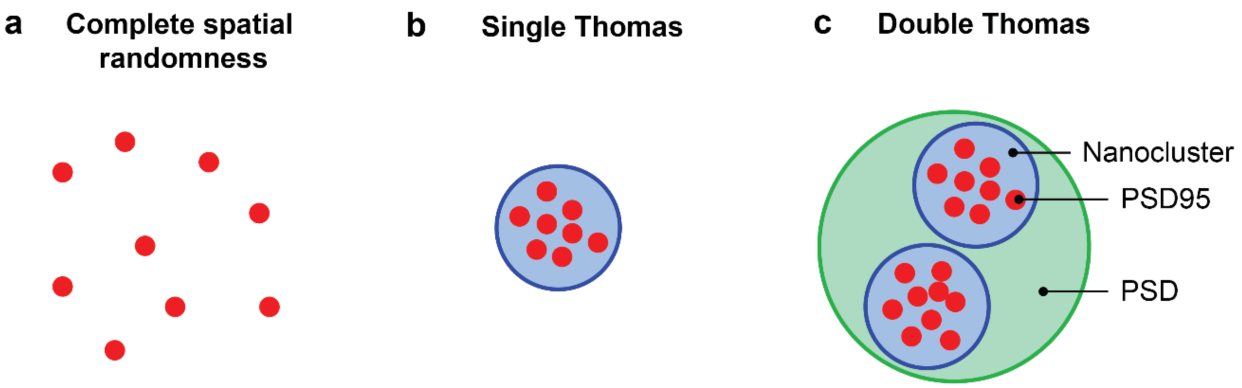

Figure S7. Pair correlation fit analysis schematic. Three different methods of clustering analysis are presented. **a** Localisations are completely spatially random. **b** Single Thomas process outputs nanocluster size and the extent of clustering. **c** Double Thomas process outputs the size of a nanocluster, a PSD, and the extent of clustering for each.

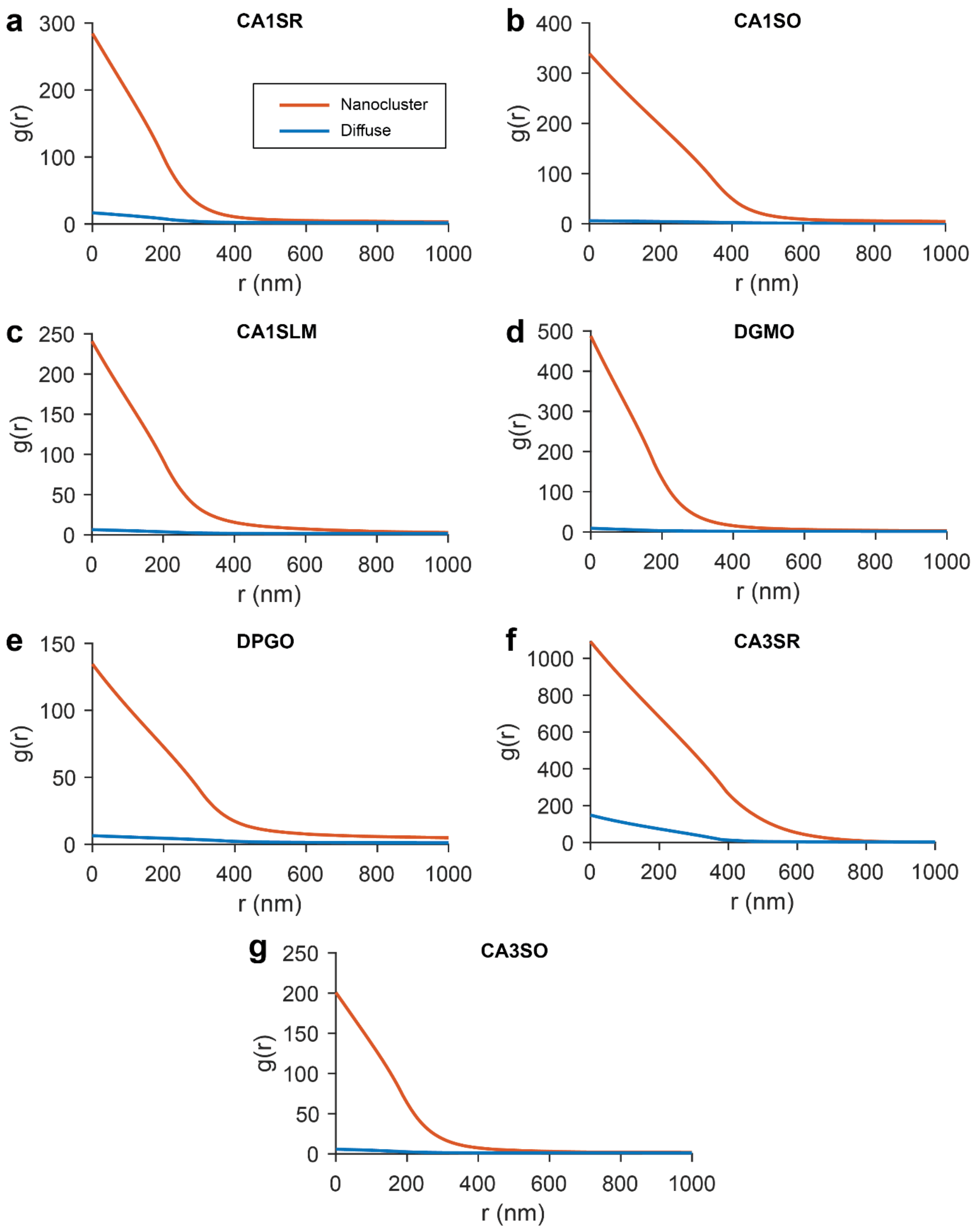

Figure S8. Pair correlation of diffuse and nanocluster populations in different brain regions. PCF fits for each hippocampal sub-region showing diffuse (blue) and NC (orange) fittings using single Thomas fitting. **a** CA1SR **b** CA1SO **c** CA1SLM **d** DGMO **e** DGPO **f** CA3SR **g** CA3SO.

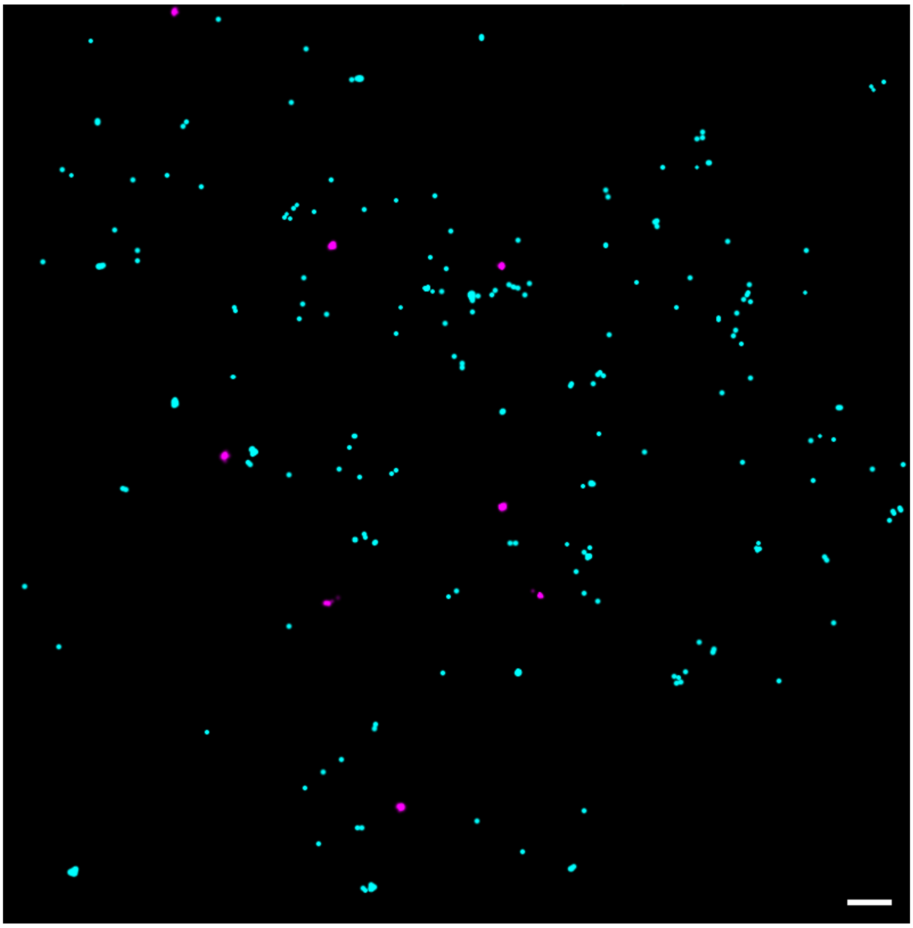

Figure S9. 2D PALM images of PSD95-mEoS2 localizations in murine brain tissue. Clustered (magenta) and non-clustered localizations (cyan). Data were generated using the Peak Fit (GDSC SMLM, ImageJ plug-in) to output localizations of the individual fluorophores. A signal strength threshold of 20 and precision threshold of 40 nm were used. A custom written Python script was used to implement a DBSCAN algorithm classify localizations into clusters, with parameters ε and minimum localizations set to 1.0 and 10 respectively. A total of 2753 localizations were clustered and 5546 non-clustered, indicating an extra-synaptic population of around 70%. Scale bar 500 nm.

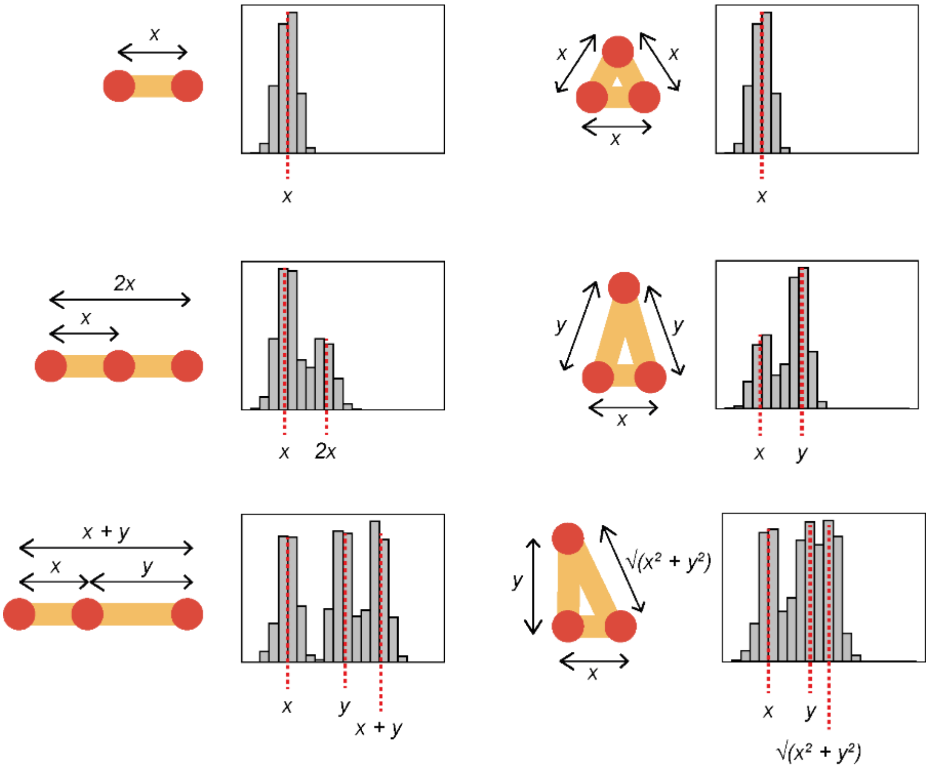

Figure S10. Schematic showing possible geometries of PSD95 clusters that would produce two distinct distance populations. None of these or higher order geometries represent the data we collected. Therefore, there must be two populations of dimeric clusters with distinct separation distances.

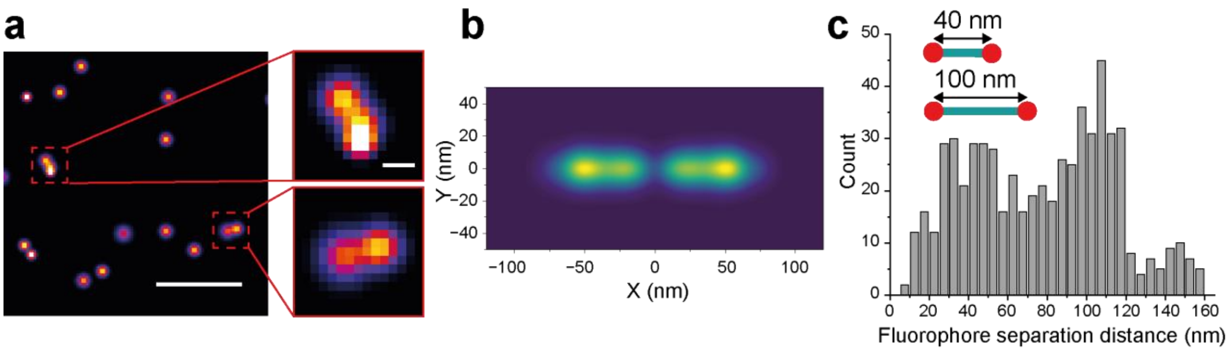

Figure S11. Identification of dimers in PSD95 endogenously fused to HaloTag and labelled with photoactivatable Janelia Fluor 549 (PAJF549). a Example PALM image of PSD95 endogenously tagged with HaloTag conjugated to PAJF549 in tissue sections from the CA1 region of the hippocampus. Scale bar 400 nm, inset scale bar 40 nm. **b** The class average of 602 dimeric objects is shown. **c** Histogram of separation distances between the dimeric PSD95 clusters seen in tissue sections.

S3: Supporting Movies

SI Movie 1 | PSD95-mEos2: Raw DHPSF localization data.

Representative raw single-molecule localisation data. An exposure time of 50 ms was used with power densities of 0.25 kW cm^{-2} and 0.7 W cm^{-2} for the 561 and 405 nm laser lines, respectively.

SI Movie 2 | PSD95-mEos2: Diffraction-limited vs. super-resolved.

Representative diffraction-limited image vs. super-resolution image of PSD95-mEoS2 taken from the CA1SR region.

SI Movie 3 | PSD95-mEos2: Clusters and supercomplexes.

Shows a representative super-resolution reconstruction of PSD95-mEoS2 taken from the CA1SR. The movie rotates around a central axis. Initially the reconstruction is shown with a density threshold applied to accentuate the PSD95 nanoclusters, this makes larger clusters more obvious and is similar to the analysis applied in our previous study. Halfway through the movie we switch to a rendering that shows all localisations (show in red). These data support the findings in Fig 3c that the vast majority of localisations do not reside in the NCs.

References

(1) Kovačević, N.; Henderson, J. T.; Chan, E.; Lifshitz, N.; Bishop, J.; Evans, A. C.; Henkelman, R. M.; Chen, X. J. A Three-Dimensional MRI Atlas of the Mouse Brain with Estimates of the Average and Variability. *Cereb. Cortex* **2005**, *15* (5), 639–645. https://doi.org/10.1093/cercor/bhh165.

(2) Santuy, A.; Tomás-Roca, L.; Rodríguez, J.-R.; González-Soriano, J.; Zhu, F.; Qiu, Z.; Grant, S. G. N.; DeFelipe, J.; Merchan-Perez, A. Estimation of the Number of Synapses in the Hippocampus and Brain-Wide by Volume Electron Microscopy and Genetic Labeling. *Sci. Rep.* **2020**, *10*, 14014. https://doi.org/10.1038/s41598-020-70859-5.

(3) Zhu, F.; Cizeron, M.; Qiu, Z.; Benavides-Piccione, R.; Kopanitsa, M. V.; Skene, N. G.; Koniaris, B.; DeFelipe, J.; Fransén, E.; Komiyama, N. H.; Grant, S. G. N. Architecture of the Mouse Brain Synaptome. *Neuron* **2018**, *99* (4), 781-799.e10. https://doi.org/10.1016/j.neuron.2018.07.007.

(4) Hirabayashi, A.; Fukunaga, Y.; Miyazawa, A. Structural Analysis of the PSD-95 Cluster by Electron Tomography and CEMOVIS: A Proposal for the Application of the Genetically Encoded Metallothionein Tag. *Microsc. Oxf. Engl.* **2014**, *63* (3), 227–234. https://doi.org/10.1093/jmicro/dfu006.

(5) Carr, A. R.; Ponjavic, A.; Basu, S.; McColl, J.; Santos, A. M.; Davis, S.; Laue, E. D.; Klenerman, D.; Lee, S. F. Three-Dimensional Super-Resolution in Eukaryotic Cells Using the Double-Helix Point Spread Function. *Biophys. J.* **2017**, *112* (7), 1444–1454. https://doi.org/10.1016/j.bpj.2017.02.023.

(6) Bulovaite, E.; Qiu, Z.; Kratschke, M.; Zgraj, A.; Fricker, D. G.; Tuck, E. J.; Gokhale, R.; Koniaris, B.; Jami, S. A.; Merino-Serrais, P.; Husi, E.; Mendive-Tapia, L.; Vendrell, M.; O'Dell, T. J.; DeFelipe, J.; Komiyama, N. H.; Holtmaat, A.; Fransén, E.; Grant, S. G. N. A Brain Atlas of Synapse Protein Lifetime across the Mouse Lifespan. *Neuron* **2022**, *110* (24), 4057-4073.e8. https://doi.org/10.1016/j.neuron.2022.09.009.
